# Trauma induced clotting factor depletion in severely injured children: a single center observational study

**DOI:** 10.1186/s13017-020-00311-6

**Published:** 2020-05-06

**Authors:** Manuel Burggraf, Christina Polan, Martin Husen, Bastian Mester, Alexander Wegner, Daniel Spodeck, Marcel Dudda, Max Daniel Kauther

**Affiliations:** 1 Department of Trauma, Hand and Reconstructive Surgery, University Hospital Essen, University of Duisburg-Essen, Hufelandstr. 55, 45147 Essen, Germany; 2 Department of Orthopedics and Trauma Surgery, University Hospital Essen, University of Duisburg-Essen, Hufelandstr. 55, 45147 Essen, Germany

**Keywords:** Coagulation, Trauma, Clotting factors, Coagulopathy, Children

## Abstract

**Background:**

Coagulopathy following severe trauma contributes significantly to mortality. Impaired clotting factors have been observed in adult trauma patients, but in pediatric trauma victims their activity has not yet been investigated.

**Methods:**

Sixteen pediatric trauma patients were evaluated according to the ISS and assigned to two cohorts. An additional control group (CO; *n* = 10) was formed. Routine coagulation parameters and the soluble clotting factors (F) were tested. Nonparametric data was analyzed using the Mann-Whitney *U* test. Results are reported as median and interquartile range.

**Results:**

The ISS of severely (SI, *n* = 8) and mildly (MI, *n* = 8) injured children differed significantly (25 [19–28] vs. 5 [4–6]; *p* < 0.001). INR was elevated in the SI cohort only when compared to the CO (1.21 [1.04-1.58] vs. 0.96 [0.93-1.00]; *p* = 0.001). Differences between SI and MI were found for FII (67 [53-90] vs. 82 [76-114] %; *p* = 0.028), FV (76 [47-88] vs. 92 [82-99] %; *p* = 0.028), and FXIII (67 [62-87] vs. 90 [77-102] %; *p* = 0.021). Comparison of the SI with the CO (FII 122 [112-144] %; *p* < 0.001; FV 123 [100-142] %; *p* = 0.002; and FXIII 102 [79-115] %; *p* = 0.006) also revealed a reduction in the activity of these factors. Furthermore, fibrinogen (198 [80-242] vs. 296 [204-324] mg/dl; *p* = 0.034), FVII (71 [63-97] vs. 114 [100-152] %; *p* = 0.009), FIX (84 [67-103] vs. 110 [90-114] %; *p* = 0.043), and FX (70 [61-85] vs. 122 [96-140] %; *p* = 0.001) were reduced in the SI in comparison with the CO. Finally, FVIII was considerably, yet not significantly, increased in both patient cohorts (235 [91-320] % and 197 [164-238] %, respectively).

**Conclusions:**

This study proves that children suffer a depletion of clotting factors following severe injury which basically reflects the findings for adult trauma patients. Attempts to correct the impaired clotting factor activity could be based on a specific hemostatic therapy involving administration of coagulation factors. Nevertheless, therapeutic implications need to be investigated in future studies.

## Background

Severe trauma is one of the most commonly reported causes of mortality not only in adults, but especially during childhood and adolescence [1–3]. Besides traumatic brain injury (TBI), severe bleeding is the leading cause of casualties in adult trauma patients, and accompanying coagulopathy on admission is clearly correlated to mortality ([4–6]. Trauma-induced coagulopathy (TIC) differs significantly from other forms of coagulation disorders such as disseminated intravascular coagulation [7]. In recent years, the particular significance of TIC has also been confirmed for pediatric trauma patients in civilian and combat support hospitals [8–10]. Hence, errors in the clinical management of bleeding remain a major issue. They account for the majority of potentially preventable deaths in both adult and pediatric trauma victims and are at least partially due to improper correction of devastating coagulopathies [11–13]. In this context, early detection of TIC is still a challenge as routine coagulation tests such as prothrombin time (PT) have known limitations [14]. Furthermore, though pathological PT values are predictive of an unfavorable outcome, they are not necessarily indicative of coagulopathy or bleeding in injured children [15].

These days it is known that traumatic coagulopathy is a multifactorial process, yet the underlying mechanisms leading to TIC are still not fully understood. However, dilution and/or consumption of clotting factors are an important driver in its development [16]. An early study by Harrigan et al. demonstrated a depletion of some clotting factors for patients in severe hemorrhagic shock shortly after injury [17]. In fact, several studies confirmed the decrease in coagulation factor levels or activity following severe multiple trauma in adult patients [18–23]. Nevertheless, data about clotting factor profiles for injured children is currently sparse. In a recent study using a method called principal component analysis, Leeper et al. showed that the component representing global depletion of clotting factors is linked to mortality and early transfusion in children as well [24]. However, there is still no direct evidence for the derangement of clotting factors and its potential distribution among the particular coagulation factors in a pediatric trauma cohort.

Therefore, the aim of this study was to further elucidate potential alterations in the soluble clotting factors at an early stage in order to form a rational basis for empiric coagulation therapy in severely injured children.

## Methods

An observational study design was chosen, and all data was collected prospectively. Children with multiple injuries under the age of 18 years who met the highest-level trauma activation criteria and had been primarily admitted from the scene of the accident to the trauma resuscitation room of our institution (level 1 Trauma Center in Germany) were screened for enrollment. Exclusion criteria comprised age ≥ 18 years, known coagulation disorders, anticoagulant medication, pregnancy, or transfer from another hospital. Enrolled children were then classified according to the injury severity score (ISS) and two distinct patient cohorts were formed. Children with an ISS ≥ 16 points were considered as severely injured (SI), those with an ISS < 16 as mildly injured (MI). Additionally, ten healthy adult donors formed a control group (CO). An adult control was chosen for ethical reasons, as otherwise, blood specimens would have had to be taken from healthy and in some cases very young children. In this context, significant differences in hemostatic parameters between children and adults were found primarily only for the very young under 1 year of age [25]. The study was performed in accordance with the Declaration of Helsinki and approved by the relevant local ethics committee (reference 12-5120-BO). The parents and/or legal guardians provided informed consent.

Immediately after admission, routine samples, e.g., for blood count, and an additional citrate syringe were collected. Directly after collection, the samples were taken to the hospital laboratory. If testing was not possible right away, the samples were deep-frozen and cryo-stored at − 70 °C until analysis of the next working day. This approach is generally accepted and has been shown not to interfere with coagulation assay results [26, 27]. Apart from standard coagulation tests such as international normalized ratio (INR; reflecting PT) and partial thromboplastin time (PTT), levels of fibrinogen and calcium as well as the activity of the soluble clotting factors were analyzed. These comprise the factors (F) II, FV, FVII, FVIII, FIX, FX, FXI, FXII, and FXIII. Factor activity was assessed and given as a percentage of standard activity by comparison of the samples with standard human plasma assays of clotting factors (SHP, Dade Behring Marburg GmbH, Marburg, Germany). Finally, the differences between the SI and MI cohorts as well as the SI versus (vs.) the CO were statistically analyzed.

### Statistical analysis

IBM® SPSS® Statistics Version 25 was used to analyze the data and compile the graphs. Differences in demographic and clinical data were tested using the independent-samples *t* test (2-tailed) for age and with Fisher’s Exact test (2-sided) for gender, mechanism of injury, and survival. For nonparametric data (ISS, routine blood tests, clotting factor activity and levels), the exact significance [2 × (1-tailed)] of differences between the groups was computed using the Mann-Whitney *U* test. For all tests, *p* value < 0.05 was considered to be statistically significant. The results are reported as median and interquartile range (IQR) for nonparametric data, otherwise as mean ± standard deviation (SD), if not mentioned otherwise. The graphs show boxplots with the line across the box representing the median, the top and bottom of the box reflecting the IQR, and whiskers being defined as minimum (or 25th percentile minus 1.5 × IQR) and maximum (or 75th percentile plus 1.5 × IQR).

## Results

### Demographical data

During the study period, 17 injured children were enrolled, one of whom had to be excluded from the final analysis due to transfer from a different hospital. Based on the ISS, two cohorts of eight children each were formed (Table 1). As intended, the ISS differed significantly between the two groups (SI 25 [19–28] vs. MI 5 [4–6] points; *p* < 0.001). One child in the SI cohort died during the further course. On average, the children of the SI group were slightly but non-significantly younger than those of the MI group (9 ± 6 vs. 11 ± 5 years; *p* = 0.30). In contrast, the members of the CO were significantly older (40 ± 9 years; *p* < 0.001). One child in the SI and two in the MI group suffered from isolated TBI, whereas none sustained a penetrating injury. In fact, there were no statistically significant differences between the two groups in terms of gender, survival, or trauma mechanism.

**Table 1:** Demographic and clinical data of patient cohorts and adult controls

	SI		MI			CO		
Demographics	Mean, median, or %	SD/IQR	Mean, median, or %	SD/IQR	p*	Mean, median, or %	SD/IQR	p*
Age, year	9	6	11	5	0.30	40	9	< 0.001
Sex (male)	75.0%		62.5%		1.00	70,0%		1.00
Isolated TBI	12.5%		25.0%		1.00			
Survival	87.5%		100%		1.00			
ISS	25	19-28	5	4-6	< 0.001			
INR	1.21	1.04-1.58	1.07	1.04-1.09	0.13	0.96	0.93-1.00	0.001
PTT (sec)	26.7	24.1-34.6	26.0	25.0-28.5	0.88	28.8	26.6-31.0	0.57
Hemoglobin (g/dl)	11.8	9.9-12.6	12.9	11.5-13.6	0.11	14.9	13.8-15.6	n/a
Thrombocytes/nl	279	179-311	273	209-307	1.00	226	183-298	n/a

The demographic and clinical data of severely injured children (SI, *n* = 8) were compared with those of children with minor injury (MI, *n* = 8) and an adult control group (CO, *n* = 10). Age is given as mean value with standard deviation (SD) whereas male sex, isolated traumatic brain injury (TBI), and survival rate are demonstrated as percentages. All other variables represent median values together with interquartile range (IQR, 25th to 75th percentile)

*n/a* not applicable

*Independent-samples *t* test (age), Fisher’s Exact test (sex, isolated TBI, survival), and Mann-Whitney *U* test versus SI

Whereas none of the MI children developed a need for transfusion, two of the eight SI children had transfusion requirements within the first 24 h after admission. One child received four units of packed red blood cells (PRBC) and four units of fresh frozen plasma (FFP) as well as a hemostatic therapy with prothrombin complex concentrate (PCC), tranexamic acid, and fibrinogen. The second child received a mass transfusion with 20 PRBC and two platelet concentrates.

### Routine blood tests

Although the elevation of INR (1.21 [1.04-1.58] vs. 1.07 [1.04-1.09]; *p* = 0.13) as well as the reduced levels of hemoglobin (11.8 [9.9-12.6] vs. 12.9 [11.5-13.6] g/dl; *p* = 0.11) indicates a robust trend towards a more deteriorated condition in the SI than the MI, the analysis of these routine parameters showed no significant differences between the two groups (Table 1). Moreover, differences in PTT and thrombocyte counts were negligible. Nevertheless, INR was elevated significantly in the SI cohort compared with INR in the CO (1.21 [1.04-1.58] vs. 0.96 [0.93-1.00]; *p* = 0.001).

### Coagulation factor activity

**Fig. 1:**
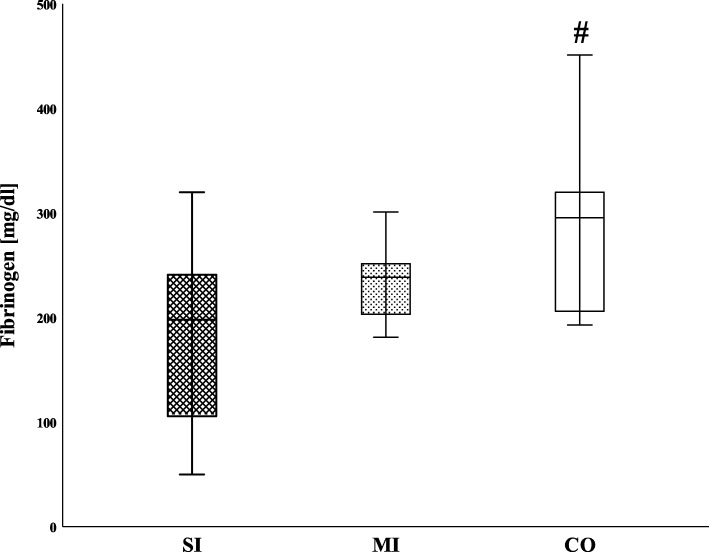
Levels of fibrinogen of injured children and controls. SI, severely injured children; MI, mildly injured children; CO, controls. ^#^*p* < 0.05 Mann-Whitney *U* test versus SI

**Fig. 2:**
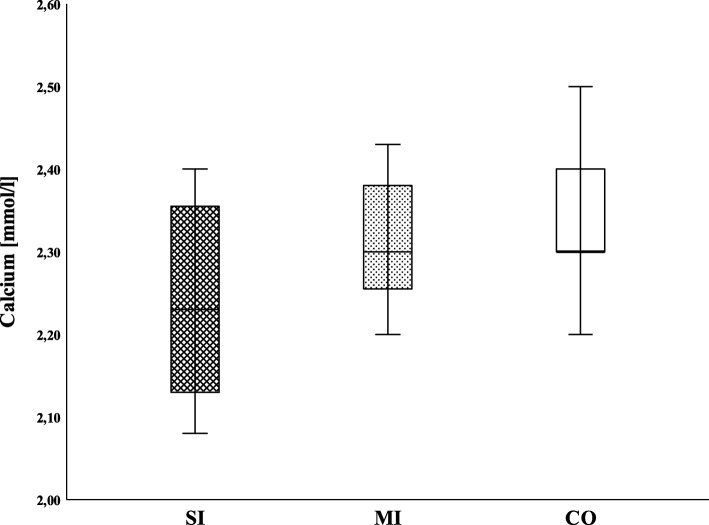
Levels of calcium of injured children and controls. SI, severely injured children; MI, mildly injured children; CO, controls

**Fig. 3:**
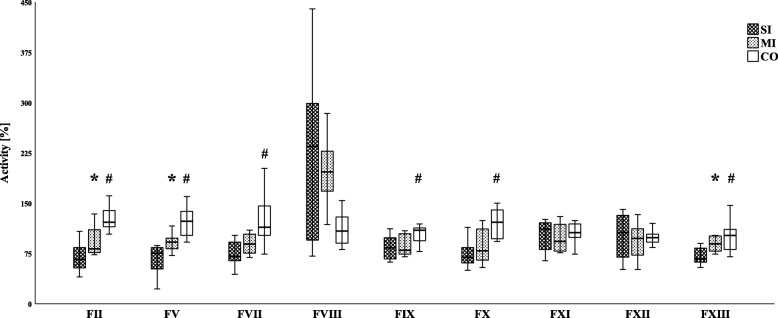
Activities of the soluble clotting factors of injured children and controls. F, clotting factor; SI, severely injured children; MI, mildly injured children; CO, controls. ^*^p < 0.05 Mann-Whitney *U* test MI versus SI; ^#^*p* < 0.05 Mann-Whitney *U* test CO versus SI

Table 2 shows the clotting factor activity for the soluble clotting factors in the SI, MI, and CO groups. The median levels of fibrinogen in the SI cohort were significantly reduced only in comparison with the CO (198 [80-242] vs. 296 [204-324] mg/dl; *p* = 0.034) (Fig. 1). Furthermore, there were no significant differences in the level of calcium between the SI and the other two groups (Fig. 2). As shown in Fig. 3, the remaining clotting factors FII and FXIII demonstrated the lowest median activity in the SI cohort with 67% [53-90 and 62-87 respectively]. This reduction was significant in comparison with the MI cohort (FII 82 [76-114] %; *p* = 0.028 and FXIII 90 [77-102] %; *p* = 0.021) as well as with the CO group (FII 122 [112-144] %; *p* < 0.001 and FXIII 102 [79-115] %; *p* = 0.006). Moreover, FV was significantly reduced in the SI in comparison with the MI patients (76 [47-88] vs. 92 [82-99] %; *p* = 0.028) and the CO group (123 [100-142] %; *p* = 0.002). Although there was an obvious trend in SI children towards a decrease in the median activity of FVII (71 [63-97] %) and FX (70 [61-85] %), this did not reach statistical significance when compared with the MI cohort (FVII 89 [75-107] %; *p* = 0.16 and FX 79 [65-115] %; *p* = 0.33). Hence, when compared with the CO, the decline was statistically significant (CO FVII 114 [100-152] %; *p* = 0.009 and CO FX 122 [96-140] %; *p* = 0.001). In addition, when comparing the SI and CO, FIX was significantly reduced in the SI cohort (84 [67-103] % vs. 110 [90-114] %; *p* = 0.043). Finally, the median activity of FVIII showed a considerable increase in both patient cohorts (SI 235 [91-320] % and MI 197 [164-238] %, respectively) compared with the CO, but although a strong trend was identified between the SI and CO (108 [89-133] %; *p* = 0.17), no significant differences were detected.

**Table 2:** Clotting factor activity and levels in patient cohorts and adult controls

	SI		MI			CO		
Activity/level	Median	IQR	Median	IQR	p*	Median	IQR	p*
Fibrinogen (mg/dl)	198	80-242	239	195-253	0.20	296	204-324	0.034
FII	67	53-90	82	76-114	0.028	122	112-144	< 0.001
Ca (mmol/l)	2.23	2.13-2.36	2.30	2.25-2.40	0.38	2.30	2.30-2.40	0.36
FV	76	47-85	92	82-99	0.028	123	100-142	0.002
FVII	71	63-97	89	75-107	0.16	114	100-152	0.009
FVIII	235	91-320	197	164-238	0.72	108	89-133	0.17
FIX	84	67-103	80	73-107	0.72	110	90-114	0.043
FX	70	61-85	79	65-115	0.33	122	96-140	0.001
FXI	112	80-122	93	77-119	0.72	106	97-119	0.97
FXII	107	66-132	98	67-117	0.65	99	90-104	0.90
FXIII	67	62-87	90	77-102	0.021	102	79-115	0.006

The clotting factor profiles of severely injured children (SI, *n* = 8) were compared with those of children with minor injury (MI, *n* = 8) and an adult control group (CO, *n* = 10). Coagulation factor activity is given as a percentage, except for fibrinogen and calcium (Ca). All variables represent median values together with interquartile range (IQR, 25th to 75th percentile)

*Mann-Whitney U test versus SI

## Discussion

To the best of our knowledge, this is the first study investigating the full range of soluble coagulation factors following pediatric trauma. In comparison with the MI, the activity of FII, FV, and FXIII in the SI cohort was significantly reduced. In addition to these factors, the activity of fibrinogen as well as FVII, FIX, and FX was lower in the SI in comparison with the CO. Overall, the data mainly suggests a deterioration in the so called extrinsic and, in particular, the common pathway of coagulation [28]. In principal, this reflects findings in adult trauma cohorts, with minor variations in the distribution of the depletion [18–22]. For instance, whereas none of these previous studies showed a substantial reduction in FXIII (the fibrin stabilizing factor), in the present study it exhibited, together with FII, the lowest median activity among all clotting factors in the SI children. The activity of FVIII was considerably elevated in both pediatric patient cohorts compared with the CO, a result which is comparable with previous studies, though it did not reach statistical significance in this study [19–22, 29]. In contrast, the predominantly mild hypocalcemia described previously for severe trauma patients was not observed [30]. Nonetheless, as described for adult trauma victims, the activity of many of the clotting factors was reduced, but the reduction in each factor, even in the SI cohort with a relevant trauma load corresponding to an ISS of 25 points on average, was not very great [21, 22]. Indeed, only the activity of FII in the SI children lies below the laboratory reference range for the ages of 1 year and older. However, these reference ranges were primarily established to detect single factor deficiencies in hemophilia rather than a potentially pathological phenotype of combined coagulation factor deficiencies in TIC. Therefore, interpretation and action based on the observed alterations remain a challenge.

Regarding INR and PTT, these conventional coagulation tests have known shortcomings in predicting clotting factor depletions, especially those of the intrinsic and extrinsic pathways [23]. Furthermore, neither PT (and INR, accordingly) nor PTT can monitor deficiencies in FXIII activity [28]. In the present study, PTT was not at all indicative of the presence of the observed clotting factor deficiencies in the SI cohort. As confirmed by the literature, INR in general seems to be a better predictor of clotting factor deficiencies [31]. This is also in line with previously published data on pediatric TIC, showing a higher proportion of abnormal PT values (reflecting INR) compared with PTT [8, 29]. Regarding the lack of difference in INR between SI and MI, it should be noted that the INR represents the coagulation system only partially. Besides the common pathway, it is specific for the so-called extrinsic pathway in view of the classical coagulation cascade. Since no statistically significant differences between SI an MI were found for FVII, the factor that defines the extrinsic pathway, this could ultimately explain the missing difference in INR between MI and SI. In this context, the use of viscoelastic assays (thromboelastography and rotation thromboelastometry, respectively) as point-of-care procedures has proven to be beneficial and we advocate its use [14]. However, these viscoelastic tests are still infrequently used in the care of pediatric trauma patients, even in highly developed trauma centers [32].

With regard to the elevated FVIII activity, which in both patient cohorts lay above the upper limit of the given reference range, the most plausible reason for this is an acute phase reaction [33]. The potential implications have been discussed in detail previously [22]. Most importantly, the increase in FVIII activity might interfere with in-vitro PTT measurement, leading to false-negative results [31]. This could at least partially explain the above statements on the role of PTT in trauma. Furthermore, as the elevated FVIII correlated with the results of the viscoelastic tests, this might also have an undesirable effect on the latter [29, 34]. Additionally, the increase in FVIII activity might, at least partially, explain the phenomenon of trauma-induced hypercoagulability [29]. With regard to the possibly far-reaching consequences, all of this should be investigated in future studies.

Regarding the therapeutic implications of this study, when attempting to actively correct clotting factor activity to normal values rather than to prevent further dilution, FFP alone might be not ideal. Coagulation factor levels in FFP are somewhat inconsistent and usually do not correct coagulation factor deficiencies sufficiently when given at a standard dosage in critically ill adults [35]. Comparably, FFP regularly fails to substantially correct abnormal INR values in children with critical bleeding and, if at all, only in those with severe deterioration (INR > 2.5) [36]. Indeed, though the implementation of a massive transfusion protocol with high ratios of FFP seems to be feasible in coagulopathic pediatric trauma victims, a beneficial effect on survival could not be demonstrated [37]. In contrast, several reports show promising results for specific coagulation factor-based resuscitation in pediatric and adult trauma patients [38]. This study provides a rationale for this, as a series of crucial components in the coagulation cascade seem to be moderately affected, and hemostatic agents containing higher ratios of clotting factors might be warranted to restore normal coagulation. Four-factor PCC could be such an agent, as it contains FII, FVII, FIX, and FX in a concentration up to 30 times higher than standard FFP [38]. Moreover, as FVII activity was reduced in the SI compared with the CO, there might be a potential value of recombinant factor VIIa (rVIIa) administration in pediatric trauma patients. Indeed, there has been a positive report on the beneficial application of rVIIa in a severely injured child [39]. Hence, in the subgroup analyses of two studies on the off-label use of rVIIa in pediatric patients no survival benefit could be demonstrated and severe thromboembolic complications were observed [40, 41]. All in all, specific hemostatic therapy based on viscoelastic tests as described by Schochl et al. for adult trauma patients might be the most favorable treatment option for coagulopathic pediatric trauma victims [42].

## Limitations

This study has several limitations. Firstly, it is an observational study and both cohorts comprise only a small number of patients. Therefore, this study is prone to various types of bias [43]. Nevertheless, the results were clearly in line with the findings of comparable studies investigating adult trauma patients, thus we assume the data of this study to be reliable in general [19–22]. Secondly, it is not a mechanistic study and we cannot make conclusions about the causality leading to reduced levels and activity of some of the clotting factors. In particular, this study cannot discriminate between endogenous (protein C pathway activation) and exogenous (depletion and/or dilution) contributors as described by Christiaans et al. [38]. Regarding patient composition, clotting factor depletion seems to be related especially to penetrating injury [44]. As penetrating trauma was absent in both cohorts of this study, this could possibly impair the generalizability of the findings. The intertwined underlying mechanisms leading to and resulting in coagulopathy and massive bleeding might differ between penetrating and blunt trauma. Nevertheless, blunt trauma seems to be the predominant type of injury in children in a civilian setting, in contrast to a military conflict environment [9, 45]. Additionally, the ISS was chosen to establish the two patient cohorts as it has been shown that a high ISS is closely linked to coagulopathy in pediatric patients [45]. Mathematically, one-quarter of the SI children in this study had transfusion requirements, which corresponds to the observed transfusion rate for severely injured adults [46].

Furthermore, it would have been interesting to evaluate the subgroup of children suffering from severe (isolated) TBI, as it is known that pediatric TBI might act as a trigger for coagulopathy, thus potentiating the stand-alone lethal effects leading to further increased morbidity and mortality [45, 47, 48]. However, due to the fact that there was only one such child in the already small SI cohort, this was not feasible. Therefore, the particular impact of TBI on clotting factor disturbances has to be elucidated in larger studies.

The decision to apply an adult control group could be an important limitation as it interferes with the concept of developmental hemostasis as proposed by Andrew [49]. Nevertheless, it was recently confirmed that most laboratory tests for hemostatic parameters do not show significant differences between the median values of adults and children over the age of 1 year [25]. In fact it was shown that PT (reflecting INR) and FV are comparable in adults and children throughout all age groups. In contrast, PTT was longer until the end of the first year of life. The plasma levels of all clotting factors (except FV) are reduced most of the time during that early period in life. After the first year of life, differences are sparse and comprise reduced levels of fibrinogen up to the age of 5 years. In fact, only one child in the SI cohort (and none in the MI group) was younger than 1 year of age. Interestingly, a study comparing the reference values of healthy children, including those under the age of 1 year, and adults did not find any significant differences between the two groups in a viscoelastic test [50]. Most importantly, the differences observed between the SI and the CO in this study are much greater than the differences in the reference values described above [25]. In summary, there is conclusive evidence that the respective potential bias is rather negligible and we therefore decided to apply a study design without a pediatric control group. In contrast, hemoglobin and thrombocyte count only serve as orientation values, as reference ranges vary considerably depending on age and gender. Consequently, we decided not to test for statistical difference between the SI and the CO, also because differences between these two parameters were not within the specific scope of this study.

Finally, coagulation is obviously not only built on the soluble coagulation factors. Cellular components also play a crucial role. Indeed, this study basically evaluates the components of secondary and partially tertiary, not primary hemostasis, which is based on platelets to a significant degree [28]. In the context of trauma, platelet function is an important, yet often overlooked factor about which this study cannot draw any conclusions [51].

## Conclusions

In conclusion, this study demonstrated a moderate but widespread depletion of clotting factors following severe pediatric trauma. Mainly the extrinsic and common pathways of the coagulation cascade were affected. This basically reflects the findings for adult trauma patients reported in the literature. Likewise, FVIII activity is obviously elevated, potentially affecting the validity of PTT measurements. Attempts to correct the impaired clotting factor activity could be based on a specific hemostatic therapy involving the administration of coagulation factors. Nevertheless, therapeutic implications need to be investigated in larger prospective studies and randomized controlled trials.

## Data Availability

The datasets used and/or analyzed during the current study are available from the corresponding author on reasonable request.

## Abbreviations

CO: Controls
F: Factor
FFP: Fresh frozen plasma
INR: International normalized ratio
IQR: Interquartile range
ISS: Injury severity score
MI: Mildly injured
PCC: Prothrombin complex concentrate
PRBC: Packed red blood cells
PT: Prothrombin time
PTT: Partial thromboplastin time
SD: Standard deviation
SI: Severely injured
TBI: Traumatic brain injury
TIC: Trauma-induced coagulopathy
